# Induction of Smooth Muscle Differentiation in Fibroblasts by Modulation of Cytoplasmic Actin Ratio

**DOI:** 10.3390/ijms27135820

**Published:** 2026-06-27

**Authors:** Yulia Levuschkina, Vera Dugina, Galina Shagieva, Anton Burakov, Dmitry Kudlay, Sergei Boichuk, Radik Faskhutdinov, Svetlana Vinokurova, Natalia Khromova, Pavel Kopnin

**Affiliations:** 1 A.N. Belozersky Institute of Physico-Chemical Biology, Lomonosov Moscow State University, 119991 Moscow, Russiadugina@belozersky.msu.ru (V.D.);; 2 Biological Faculty, Lomonosov Moscow State University, 119991 Moscow, Russia; 3 Department of Pharmacognosy and Industrial Pharmacy, Lomonosov Moscow State University, 119992 Moscow, Russia; 4 Department of Pharmacology, The I.M. Sechenov First Moscow State Medical University (The Sechenov University), 119991 Moscow, Russia; 5 Department of Pathology, Kazan State Medical University, 420012 Kazan, Russia; 6 Department of Radiotherapy and Radiology, Russian Medical Academy of Continuous Professional Education, 119454 Moscow, Russia; 7 N.N. Blokhin National Medical Research Center of Oncology, 115478 Moscow, Russia

**Keywords:** actin isoforms, myogenic differentiation, fibroblasts, smooth muscle cells, trans-differentiation

## Abstract

Myogenic differentiation is a powerful mechanism for generating diverse cell types from fibroblasts. Here, we show that targeted suppression of β-actin by RNA interference in human fibroblasts triggers coordinated molecular and structural changes consistent with trans-differentiation toward SMC-like phenotype. This conversion is marked by upregulation of smooth muscle differentiation markers (α- and γ-smooth muscle actins, SM22, smooth muscle myosin, desmin, vinculin) at mRNA and protein levels, together with distinct morphological alterations: increased cell area, loss of polarity, and reorganization of the actin cytoskeleton. Notably, β-actin-downregulated fibroblasts exhibited a focal adhesion architecture that differed from parental fibroblasts. These findings indicate that β-actin downregulation may provide a novel *in vitro* method to induce SMC-like differentiation, with potential implications for vascular biology and tissue engineering.

## 1. Introduction

Fibroblasts have the capacity to differentiate into a number of other cell types, including myofibroblasts and smooth muscle cells (SMCs). In previous studies, we have demonstrated the potential for induced differentiation of fibroblasts into myofibroblasts through the suppression of γ-cytoplasmic actin (hereafter referred to as γ-actin) expression [1]. Further research into the effects of altering the cytoplasmic actin ratio; in particular, the downregulation of β-actin in the present study could provide fundamental insights into the mechanisms of trans-differentiation and the derivation of new cell types. The actin cytoskeleton is involved in numerous intracellular processes and influences multiple signaling pathways, so modifying it may reproduce similar transformations in vivo in a more complete manner than using differentiation factors in experimental culture treatments. The structural differences between the various actin isoforms are extremely insignificant at the protein level. When considering the N-terminal region of β- and γ-actin, a mere four positions are evident [2]. Usually, γ-actin assembles into a branched cortical network, while β-actin forms stress fibers located on the ventral side of the cell [3]. Consequently, the effects of these isoforms on cell signaling pathways are also determined by their divergent affinities for regulatory factors, including MRTF-A, MyoD, and others [4,5]. Various studies on the epigenetic regulation of myogenesis have focused on the role of the H3 and H2A histones in attracting transcriptional regulators to myogenic loci [6,7].

Vascular SMCs are represented by a set of phenotypic classes that differ from each other primarily in terms of cell shape, certain cytoskeletal markers and the ratio of muscle actin isoforms [8]. Mature vascular SMCs are characterized by a predominantly contractile phenotype. However, in the event of vascular injury, contractile SMCs undergo a transformation into synthetic SMCs, a process that can be compared to dedifferentiation. During this process, SMCs lose their contractile abilities and acquire the active proliferation, migration and synthetic capacities characteristic of the synthetic phenotype [9]. While this plasticity undoubtedly benefits the survival of the organism, failure to revert to the contractile phenotype can lead to neointimal hyperplasia, contributing to various pathological processes [9]. In this context, contractile SMCs are increasingly recognized as the primary objects of study, while synthetic SMCs are the focus point for the development of therapeutic strategies against atherosclerotic conditions.

The identification of SMCs in tissue structures is difficult, mainly due to the lack of differentiation markers that can distinguish them from the adjacent cell types, or reflect the degree of differentiation and phenotypic features of SMC subtypes. The α-Smooth muscle actin (α-SMA) is the most commonly used SMC marker due to its commercial availability and detection efficiency, but this protein is also found in myofibroblasts, pericytes and other cell types [10]. Conversely, in certain pathological transformations, SMCs may change into different subtypes of synthetic SMCs and lose typical markers of the contractile apparatus [11]. Therefore, a more reliable approach to identify SMCs is to use several markers simultaneously. Additionally, classical histological methods and the analysis of ultrastructural elements, such as the presence of dense bodies in the contractile apparatus, localization in proximity to endothelial or epithelial cells and a spindle-shaped morphology, should also be considered [12].

Compared to animal models and primary cultures of human cells, SMCs derived from the induced differentiation of human fibroblasts offer an unlimited, immunocompatible source of cells, free from ethical concerns. At the same time, methods of induction from pluripotent stem cells result in a highly heterogeneous cell population. Trans-differentiation eliminates the requirement for an intermediate pluripotent state, thus representing a more efficient and faster method of obtaining a specific cell type.

## 2. Results

### 2.1. The Suppression of β-Actin Expression Alters Cell Morphology and the Ratio of Different Actin Isoforms and Leads to a Reorganization of the Contractile Apparatus

**Figure 1:**
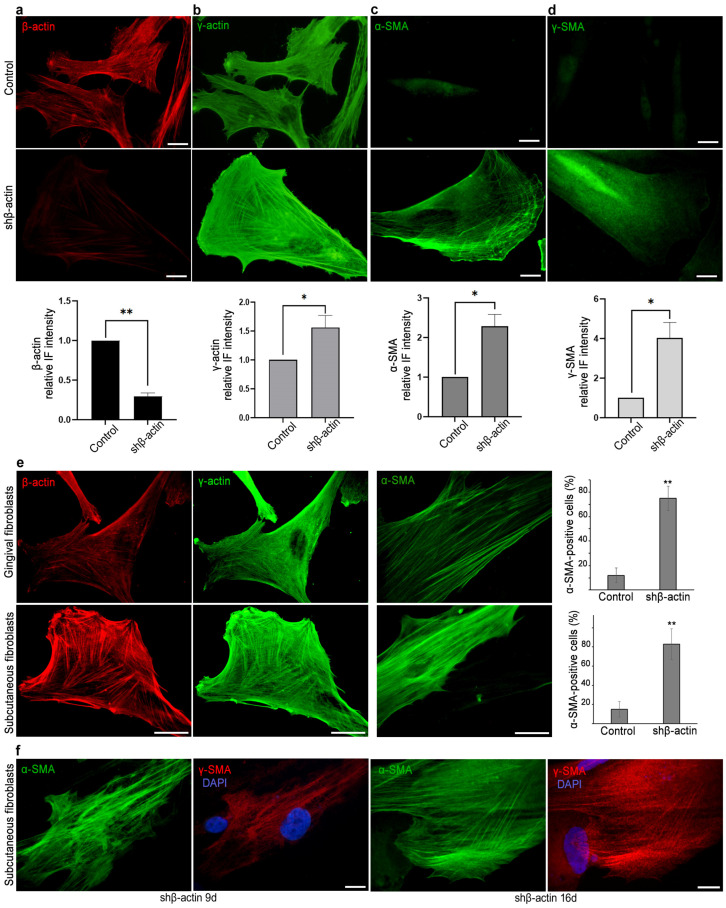
β-Actin suppression alters the expression of cytoplasmic and smooth muscle actin isoforms in human fibroblasts. (**a**–**d**) Expression of actin isoforms in control culture and culture after suppression of β-actin (gingival fibroblasts, 9 days): (**a**) β-actin, (**b**) γ-actin, (**c**) α-SMA and (**d**) γ-SMA with the mean IF intensity for each comparison. Immunofluorescence microscopy. Scale bar: 15 µm. The graphs represent analysis of IF intensity, mean ± SEM, calculated from the results of three independent experiments; at least 20 fields of view were analyzed for each biological replicate; (**e**) expression of β-actin (left), γ-actin (middle), and α-SMA (right) in control cultures of gingival fibroblasts and subcutaneous fibroblasts. Immunofluorescence microscopy. Scale bar: 25 µm. The graphs represent percentage of cells with distinct α-SMA expression in gingival (upper graph) and subcutaneous (lower graph) fibroblasts, investigated in controls and in cells with downregulated β-actin, 9 days after infection, mean ± SEM, three independent experiments, at least 20 fields of view were analyzed for each biological replicate; (**f**) expression of α-SMA and γ-SMA in subcutaneous fibroblasts after suppression of β-actin for 9 days (left) and 16 days (right). Immunofluorescence microscopy. α-SMA—green, γ-SMA—red, DAPI—blue. Scale bar: 15 µm. The Mann–Whitney U test was used for the statistical analysis of all depicted comparisons. Asterisks indicate *p*-values < 0.05 (*) or <0.01 (**) for all panels.

The method of RNA interference with short hairpin RNA (shRNA) was used to suppress the expression of the β-actin gene in human fibroblasts of two tissue origins. Two types of fibroblasts were used, as they differ in their baseline expression of smooth muscle-related markers. This provides complementary models for investigating the effects induced by β-actin downregulation. As the morphological alterations after 5–6 days were insufficient to indicate signs of smooth muscle differentiation, these intermediate differentiation periods were excluded from the study. The initial experiments were conducted on gingival fibroblasts, and the results were analyzed 9 days after infection. Staining for to cytoplasmic actin isoforms revealed a significant decrease in immunofluorescence (IF) intensity (more than 60%) for β-actin and an increase in IF intensity for γ-actin after β-actin downregulation (Figure 1a,b). In control cultures, a small number of cells exhibited IF signal for α-SMA (α-SMA-positive cells), accounting for no more than 20% of the culture. The IF intensity of both SMA isoforms increased dramatically following β-actin suppression (Figure 1c,d). The staining appeared either diffuse or in the form of poorly organized bundles. In cultures with suppressed β-actin, the proportion of α-SMA-positive cells increased significantly (Figure 1e, graphs). As a result, it was possible to distinguish various types of stress fibers that compose the actin cytoskeleton of the cells (Figure 1c, Supplementary Figure S1). The γ-SMA was not detected in the control cell culture. Meanwhile, cells exhibiting pronounced diffusely located γ-SMA expression were identified within the culture upon β-actin suppression (Figure 1d). Downregulation of β-actin altered the cellular morphology. The total cell projection area increased, the shape became less polarized, and distinct actin bundles appeared in different parts of the cell, including transverse arches, dorsal and ventral stress fibers (Figure 1a,b). Some of the morphological characteristics were assessed quantitatively in subsequent experiments (Section 2.3). It was observed that the control fibroblasts, which were obtained from different origins, exhibited significant variation in the proteins of interest. For instance, the IF of the cytoplasmic actins and α-SMA was notably higher in control subcutaneous fibroblasts than in gingival fibroblasts (Figure 1e). It was also necessary to determine the optimal differentiation time for subcutaneous fibroblasts. The most significant differences were observed in the distribution of SMA isoforms. Nine days after infection, the corresponding proteins showed predominantly diffuse distribution, whereas pronounced α-/γ-SMA bundles were observed after 16 days (Figure 1f). Manifestations similar to those observed at the considered time points (9 days and 16 days) were seen after 10–11 and 21 days, respectively.

Thus, downregulation of β-actin resulted in increased IF intensity of γ-actin, α-SMA, and γ-SMA, which was detected in both subcutaneous and gingival fibroblasts 9 days after infection and became more pronounced by day 16. These changes in IF intensity were accompanied by reorganization of the actin cytoskeleton.

### 2.2. Additional Cytoskeletal Markers Indicate a Smooth Muscle Type of Myogenic Differentiation upon β-Actin Suppression

**Figure 2:**
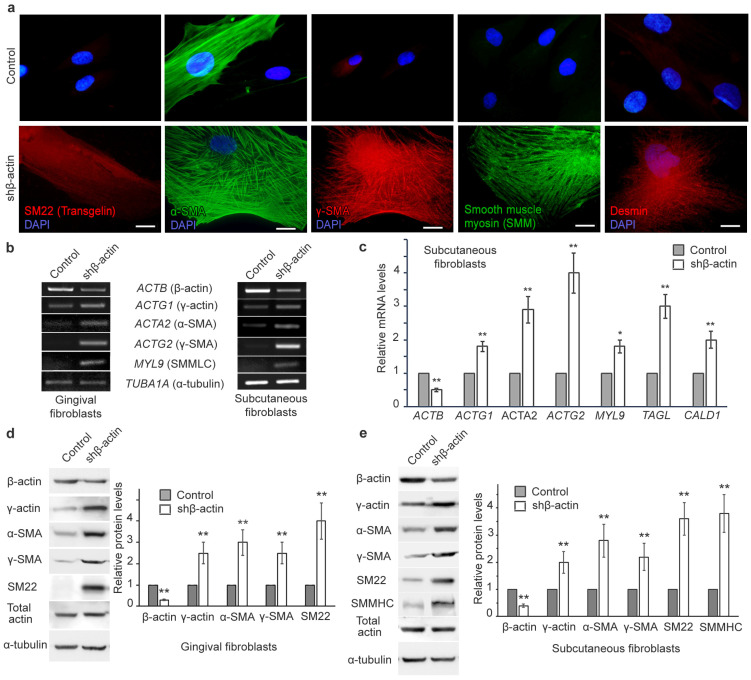
Markers of smooth muscle differentiation in human fibroblasts upon β-actin suppression. (**a**) IF staining for SM22, α-SMA, γ-SMA, SMM (smooth muscle myosin), and desmin (left to right) in human subcutaneous fibroblasts upon suppression of β-actin (16 days). Immunofluorescence microscopy. Scale bar: 15 µm; (**b**) end-point RT-PCR β-actin, γ-actin, α-SMA, γ-SMA, and smooth muscle myosin light chain (SMMLC) of human gingival (left) and human subcutaneous (right) fibroblasts (control and shRNA to β-actin, 16 days); (**c**) qRT-PCR analysis of cytoskeletal and contractile gene expression in human subcutaneous fibroblasts. Relative expression levels of *ACTB* (β-actin), *ACTG1* (γ-actin), *ACTA2* (α-SMA), *ACTG2* (γ-SMA), *MYL9* (smooth muscle myosin light chain), *TAGLN* (transgelin), and *CALD1* (caldesmon) were measured in control cells and cells subjected to β-actin downregulation (shRNA) for 16 days, mean ± SEM, calculated from the results of three independent experiments; (**d**) Western blot analysis of β-actin, γ-actin, α-SMA, γ-SMA and SM22 in human gingival fibroblasts (control and shRNA to β-actin, 16 days). (**e**) Western blot analysis of β-actin, γ-actin, α-SMA, γ-SMA, SM22, and smooth muscle myosin heavy chain (SMMHC) in human subcutaneous fibroblasts (control and shRNA to β-actin, 16 days). The graphs represent densitometric analysis of Western blots: mean ± SEM, the results of at least three independent experiments. The Mann–Whitney U test was used for the statistical analysis of all depicted comparisons. Asterisks indicate *p*-values < 0.05 (*) or <0.01 (**) for all panels.

We selected additional cytoskeletal proteins because the full range of actin isoforms alone is not sufficient for reliably determining the direction of myogenic differentiation that cells could undergo upon β-actin suppression. Some markers appear in the early stages of smooth muscle differentiation, before the SMA isoforms. Among them is SM22 (also known as transgelin) [10], which was observed in subcutaneous fibroblasts after β-actin suppression (Figure 2a).

Additional specific markers of smooth muscle differentiation were also identified, including desmin, which was distributed diffusely throughout the cell, and smooth muscle myosin (SMM), which was localized in filamentous structures and areas corresponding to the actomyosin cytoskeleton. These proteins were not detected in control cultures (Figure 2a) or upon γ-actin suppression that induced myofibroblastic differentiation [1]. It is important to note that these markers should be considered necessary, but not sufficient, when evaluated individually, since desmin is often expressed in pericytes [13] and smooth muscle myosin is expressed in myoepithelial cells [14].

The results of the end-point PCR analysis showed the changes in the balance of actin isoforms: decreased expression of β-actin was accompanied by increased expression of γ-actin and α-SMA. At the same time, an increase in the expression of markers characteristic of smooth muscle differentiation, such as γ-SMA, smooth muscle myosin light chain (SMMLC), transgelin, and caldesmon was detected (Figure 2b,c). Additional supportive RNA-seq transcriptomic data for key smooth-muscle-associated genes (*ACTA2*, *ACTG2*, *TAGLN*, *CNN2*, *MYL9*, *MYLK*, *LMOD1*, *MYH9*, *TPM2*, *VCL*) in gingival fibroblasts after β-actin downregulation are provided in Supplementary Table S1.

The results of western blotting analysis indicated an increase in the expression of γ-actin, α-SMA and γ-SMA, smooth muscle myosin heavy chain (SMMHC), early differentiation marker SM22, after β-actin suppression (Figure 2d,e; Supplementary Figure S1). Meanwhile, the total actin content remained virtually constant. A comparative western blotting analysis of both control cultures and cells with suppressed β-actin revealed that subcutaneous fibroblasts initially contain higher levels of the relevant proteins than gingival fibroblasts. The amount of additional differentiation markers in control subcutaneous fibroblasts was also higher than in gingival fibroblasts (Figure 2d,e).

### 2.3. Analysis of Cell Morphology and Focal Adhesions in Fibroblasts Before and After β-Actin Suppression

**Figure 3:**
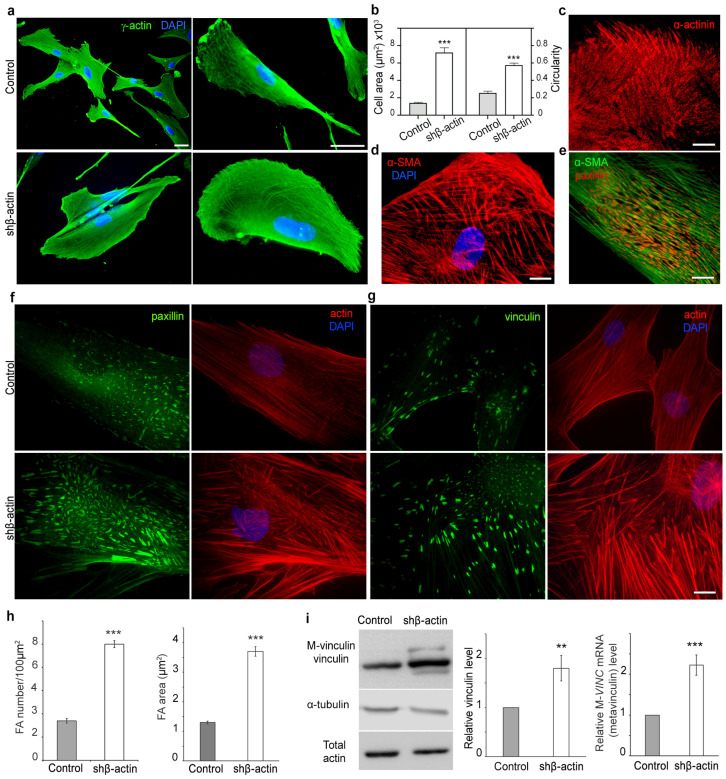
Morphometric analysis of cells before and after β-actin suppression. (**a**) The morphology of human gingival fibroblasts in control and upon β-actin suppression (9 days, two magnifications). Immunofluorescence microscopy. γ-Actin—green, DAPI—blue. Scale bar: 25 µm; (**b**) alterations in cell area and circularity of human gingival fibroblasts in control and upon β-actin suppression (9 and 16 days, mean ± SEM, three independent experiments; at least 35 cells were analyzed for each biological replicate; (**c**,**d**) identification of α-actinin-1 and α-SMA following the prolonged β-actin suppression (16 days) in human subcutaneous fibroblasts. Immunofluorescence microscopy: (**c**) α-actinin-1; (**d**) α-SMA—red, DAPI—blue. Scale bar: 15 µm; (**e**–**i**) focal adhesions (FAs) and their characteristic proteins in human subcutaneous fibroblasts (control and shRNA to β-actin, 16 days). Immunofluorescence microscopy: (**e**) α-SMA—green, paxillin—red; (**f**) actin (phalloidin)—red, paxillin—green, DAPI—blue; (**g**) actin (phalloidin)—red, vinculin—green, DAPI—blue. Scale bar: 15 µm; (**h**) analysis of the number and area of FA structures in human subcutaneous fibroblasts (control and shRNA to β-actin, 16 days). Data are presented as mean ± SEM from three independent experiments, with at least 20 fields of view analyzed for each biological replicate; (**i**) Western blot analysis of vinculin and proposed metavinculin in human subcutaneous fibroblasts (control and shRNA to β-actin, 16 days). In the right corner: qRT-PCR analysis of metavinculin expression. Data are represented as mean ± SEM, four independent experiments. The Mann–Whitney U test was used for the statistical analysis of all depicted comparisons. Asterisks indicate *p*-values < 0.01 (**) or <0.001 (***) for all panels.

To evaluate the morphometric parameters of human fibroblasts before and after suppression of β-actin, we measured total cell area and circularity. The latter parameter was calculated using the standard formula of the ImageJ plugin [15]. The suppression of β-actin resulted in an increase in the total cell area and their tendency towards circularity (Figure 3a,b). Following β-actin downregulation, the cells predominantly exhibited spindle-like or rhomboid morphologies. However, elongated cells were also observed (additional images illustrating phenotypic heterogeneity can be found in Supplementary Figure S2). IF for the actin cross-linker α-actinin-1 produced a strong, broadly distributed cytoplasmic signal consistent with its association with actin filaments along their entire length (Figure 3c). Suppression of β-actin induced the formation of thick α-SMA bundles in human subcutaneous fibroblasts (Figure 3d,e).

Alongside morphological changes, we observed a reorganization of FAs, which are essential for anchoring the actin cytoskeleton to the substrate and for contractility in SMCs. IF co-staining for α-SMA and paxillin, as well as for phalloidin and paxillin, revealed numerous FAs distributed throughout the cell projection following β-actin suppression (Figure 3e,f). Additional actin (phalloidin)/vinculin analysis indicated that a notable proportion of these FAs were vinculin-positive (Figure 3g).

Direct quantification of paxillin-positive puncta revealed increases in both the number and the area of these structures following β-actin downregulation (Figure 3f,h). Concordant results were obtained using vinculin staining (Figure 3g). Immunoblotting showed increased vinculin abundance in cells with suppressed β-actin and revealed a faint, higher-molecular-weight band consistent with metavinculin [16,17] (Figure 3i). Although this assignment is provisional and may require verification using isoform-specific antibodies, PCR analysis confirmed that metavinculin levels were elevated in human subcutaneous fibroblasts following prolonged β-actin downregulation (Figure 3i).

**Figure 4:**
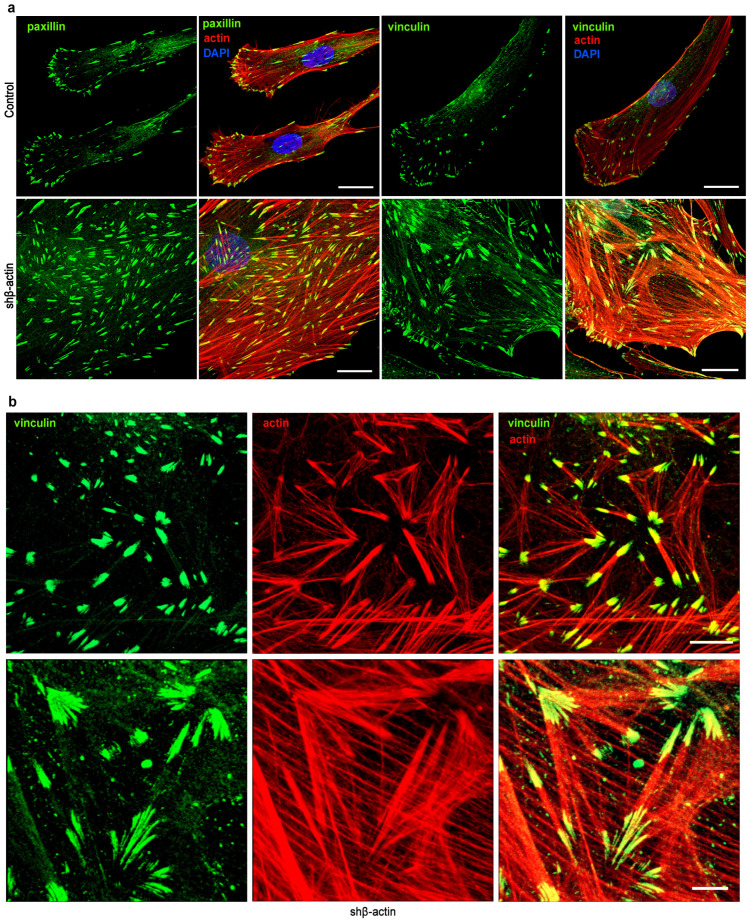
Immunofluorescence confocal microscopy of focal adhesions (FAs) in human subcutaneous fibroblasts before and after β-actin suppression. (**a**,**b**) IF staining for actin and paxillin/vinculin in FAs of human subcutaneous fibroblasts (shRNA to β-actin, 16 days). Actin (phalloidin)—red, paxillin/vinculin—green, DAPI—blue. LSM microscopy. Scale bars: 20 µm (**a**); 10 µm (upper panels) and 5 µm (upper panels) of (**b**).

We also observed redistribution of FAs after the prolonged β-actin suppression. Whereas control cells exhibited predominantly peripheral FA distribution, cells with suppressed β-actin displayed FAs throughout the lamella, including the central region rather than being restricted to the periphery (Figure 4a). In some cases, multiple actin bundles merged into larger structures that converged on FAs at different angles, or formed thick, short bundles terminated by FAs at both ends (Figure 4b).

## 3. Discussion

The process of myogenic differentiation involves the development of several specialized cell lineages, including myofibroblasts, SMCs, myoepithelial cells and pericytes. While these cell types share common cytoskeletal markers, such as α-SMA, and transcriptional regulators, like MRTF-A, they each fulfil distinct physiological functions. Our research was focused on the process of smooth muscle differentiation and the potential to induce a particular SMC phenotype.

In our study, we used fibroblasts of two tissue origins: subcutaneous fibroblasts and gingival fibroblasts. This approach was chosen due to the diversity of baseline expression of smooth muscle-related markers and parameters considered when refining the direction of induced differentiation. Subcutaneous fibroblasts initially contain some proteins that play a key role in smooth muscle differentiation (such as α-SMA, SM22, etc.), allowing them to achieve the SMC phenotype more quickly. However, gingival fibroblasts can be cultured on a substrate for longer, which enables more accurate tracking of the appearance of certain markers and assessment of FA maturation. This provides complementary models for investigating the effects induced by β-actin downregulation. We determined 9 and 16 days as the most informative time points for both fibroblast cultures, because SMA isoforms were mainly diffusely distributed 9 days after infection, whereas after 16 days, pronounced SMA bundles appeared.

The earliest observable effect of β-actin suppression in fibroblasts was an increase in the second cytoplasmic isoform of actin, γ-actin, which was evident within a relatively short differentiation period (from day 5). The temporal progression of SMA isoforms reorganization required an extended period following β-actin suppression. IF analysis showed predominantly diffuse cytoplasmic distribution on day 9, which matured into well-defined, organized contractile bundles by day 16. This time-dependent transition demonstrates the progressive nature of smooth muscle differentiation and cytoskeletal remodeling in response to altered actin isoform ratios. Although α-SMA was detectable in some control fibroblasts, γ-SMA was detected exclusively in cells after prolonged β-actin suppression. It should be noted that, at this stage, we cannot conclusively determine the precise direction of differentiation and discriminate between vascular and visceral SMC subtypes. Although α-SMA is classically associated with vascular SMCs (especially arterial SMCs), γ-SMA is the predominant actin isoform in visceral SMCs (oesophagus, stomach, intestine, bladder, and uterus) [18]. Longitudinal studies evaluating the stability of the induced phenotype and further characterizing the derived cells are required to establish whether they more closely resemble vascular or visceral SMCs.

Changes in the ratio of actin isoforms were accompanied by alterations in cell morphology, including loss of polarity, increased cell area, acquisition of a polygonal shape, and reorganization of the actin cytoskeleton. Previously, we observed that reduction of another cytoplasmic actin isoform produced a similar enlargement of fibroblasts and cell shape alteration [1]. To confirm that β-actin suppression induced smooth-muscle differentiation, we employed an expanded panel of SMC-specific markers: α-SMA, smooth muscle myosin, SM22, desmin, and γ-SMA.

FAs are large, dynamic multiprotein assemblies that also contain signaling components. These structures link the extracellular matrix to the actin cytoskeleton via integrins and mediate the transmission of mechanical force and downstream biochemical signaling, thereby playing a crucial role in cell contraction and migration [19]. We found that β-actin suppression in human fibroblasts was associated with an increase in both FA area and number, as assessed by paxillin staining. This was accompanied by the accumulation of vinculin at FAs and the appearance of a higher-molecular-weight band consistent with the vinculin isoform metavinculin, although this assignment requires verification using isoform-specific antibodies. Elevated metavinculin levels in human subcutaneous fibroblasts following prolonged β-actin downregulation were confirmed by PCR analysis. The presence of metavinculin may affect cellular contractility by altering the connection between the actin cytoskeleton and focal adhesions, which influences force transmission [16,17]. In addition to changes in abundance, we observed a clear redistribution of FAs. In control cells, FAs were predominantly localized at the lamellar edge. By contrast, β-actin suppression resulted in a significant increase in the number of FAs within the central region of the lamella rather than restricting them to the periphery. The emergence of FAs, from which multiple distinct actin bundles originated at different angles, was observed. These FAs may represent membrane-bound dense plaques [20], which are considered to be components of the smooth muscle contractile apparatus. These structural interpretations should, however, be regarded as morphological possibilities that require confirmation by electron microscopy. In summary, prolonged β-actin depletion in human subcutaneous fibroblasts induced the expression of smooth muscle actin isoforms, smooth muscle myosin, metavinculin, and leads to the reorganization of FAs, suggesting that these cells acquire altered contractility compared with control cells.

Our data indicate that β-actin downregulation triggers a coordinated program of molecular and structural changes that drive fibroblast trans-differentiation towards a smooth muscle–like phenotype. Mechanistically, we propose three overlapping pathways by which β-actin suppression promotes this transition: (1) transcriptional reprogramming via MRTF/SRF activation; (2) modulation of chromatin organization by nuclear actin; and (3) mechanotransductive changes that couple cytoskeletal forces to epigenetic regulation.

Supporting the first mechanism, our preliminary RNA sequencing analysis revealed an approximately twofold increase in *MKL2* (encoding myocardin-related transcription factor B, or MRTF-B) following β-actin downregulation. MRTF-B is essential for the in vivo development of distinct smooth muscle cell subtypes [21]. This suggests that MRTF-B/SRF signaling is involved in the observed phenotype. MRTF family proteins are regulated by the intracellular monomeric actin pool through direct binding to G-actin, which promotes their cytoplasmic sequestration [22]. As β-actin contributes substantially to the cellular G-actin pool [23], its depletion is expected to disrupt the G/F-actin balance, which is unlikely to be fully compensated by other actin isoforms. Therefore, a reduction in G-actin levels may facilitate MRTF-B nuclear accumulation and thereby enhance SRF-dependent transcription, including the activation of smooth muscle gene expression programs. Consistent with increased actin polymerization, β-actin-depleted cells exhibited more prominent phalloidin staining, indicative of elevated filamentous actin content, as phalloidin is believed to selectively bind to filamentous actin. 

Beyond MRTF/SRF signaling, our preliminary RNA-sequencing results suggest that additional pathways may contribute to the observed phenotype. In particular, *NOTCH3* was upregulated ~2-fold, consistent with its established role in smooth muscle lineage specification, and *MYLK* increased ~2.5-fold, while *NUPR1* increased ~3.5-fold. *MYLK* encodes myosin light-chain kinase, a key mediator of contractile apparatus function, and *NUPR1* has been reported to function as a transcriptional regulator promoting myogenic differentiation, suggesting that these factors may act in parallel with actin-dependent signaling to reinforce the acquisition of SMC-like characteristics.

Besides this indirect regulation via actin dynamics, actin itself can directly influence gene expression by interacting with the transcriptional machinery [23,24], chromatin-remodeling complexes [25], and overall chromatin organization [26]. Our data aligns with growing evidence that actin contributes to lineage-specific transcriptional programs in differentiating cells: osteogenic gene activation in mesenchymal stem cells [27] and heterochromatin reorganization during epidermal stem-cell commitment [28].

Several recent studies have demonstrated alternative strategies for the trans-differentiation of fibroblasts into SMC-like cells. A defined combination of transcription factors (e.g., myocardin together with MEF2C and GATA6) efficiently reprogrammed fibroblasts to SMC-like cells [29]. Human dermal fibroblasts reprogrammed with myocardin, in combination with all-trans retinoic acid (ATRA, a vitamin A derivative), acquired a functional smooth muscle phenotype with contractile properties and were effective in a mouse model of hindlimb ischemia [30]. Among the genes most upregulated in reprogrammed SMCs were those encoding actin isoforms (*ACTA1*, *ACTA2*, *ACTG2*) and *MYLK*. These studies highlight the developmental plasticity of fibroblasts. In contrast to direct transcription factor–based reprogramming, our approach relies on a subtler and more physiologically relevant perturbation: selective suppression of β-actin. Our findings provide complementary evidence that fibroblasts possess an intrinsic, latent capacity to adopt smooth muscle–like characteristics when critical cytoskeletal signaling pathways are altered.

We have previously shown that suppression of γ-cytoplasmic actin (*ACTG1*) induces differentiation of fibroblasts into myofibroblasts [1]. In the present study, downregulation of β-cytoplasmic actin (*ACTB*) results in a different differentiation trajectory: trans-differentiation towards a smooth muscle-like phenotype. Myofibroblastic differentiation generates activated fibroblasts characterized by extracellular matrix (ECM) production (e.g., collagen III and ED-A fibronectin), α-SMA expression, enlarged cell size, and prominent stress fibers. However, they lack smooth muscle markers such as SMM and desmin. In contrast, smooth muscle-like differentiation produces cells with a more developed contractile program that express not only α-SMA, but also γ-SMA, SM22/transgelin, smooth muscle myosin, desmin, vinculin/metavinculin, and that exhibit features of organized smooth muscle-type cytoskeletal architecture.

There are several limitations to the present study that should be noted. Firstly, our conclusions are predominantly based on molecular and structural markers, such as gene expression profiles and cytoskeletal organisation, rather than on functional validation of contractile properties. Secondly, the precise SMC subtype (vascular or visceral) induced by prolonged β-actin depletion is undetermined. Furthermore, we propose that the phenotype and extent of differentiation are dependent on the duration of treatment and the origin of the cell line. Finally, while our data suggest a mechanistic link between β-actin suppression and SMC differentiation, some aspects of this interpretation are preliminary.

In summary, our data demonstrate that selective downregulation of β-actin in human fibroblasts initiates a coordinated program of transcriptional and structural adaptations that collectively promote trans-differentiation towards a smooth muscle-like phenotype. This phenotype is defined by the upregulation of canonical smooth muscle markers (α-SMA, γ-SMA, SM22, and SMM), as evidenced by IF, qRT-PCR and WB analyses; additional supportive RNA-seq transcriptomic data for key smooth-muscle-associated genes (*ACTA2*, *ACTG2*, *TAGLN*, *CNN2*, *MYL9*, *MYLK*, *LMOD1*, *MYH9*, *TPM2*, *VCL*); increased cell area and altered morphology; remodeling of FAs, including increased area, paxillin/vinculin positivity, and larger FA structures with multiple actin bundles inserted at different angles. Preliminary RNA-seq evidence supports MRTF/SRF signaling as mechanistic contributor. While functional assays of contractility and migration are still required to validate the SMC identity and physiological competence of these cells, our results identify β-actin suppression as a promising experimental approach to deriving SMC-like cells from fibroblasts and provide a basis for future studies in vascular disease modelling and regenerative medicine.

## 4. Materials and Methods

### 4.1. Cell Culture and Cell Cultivation

Cell cultures of gingival and subcutaneous fibroblasts were acquired from the culture collection of the cytogenetics laboratory of the Scientific Research Institute of Carcinogenesis, N. N. Blokhin National Medical Research Center of Oncology. For the experiments, we used normal human cells at passages 4–5. The cells were maintained in Dulbecco’s Modified Eagle’s Medium (DMEM) (L0100, Biowest, Nuaillé, France) with 10% fetal bovine serum (10270-106, Gibco, Thermo Fisher Scientific, Inc., Waltham, MA, USA), GlutaMAX^TM^ (35050061, Thermo Fisher Scientific, Inc., Waltham, MA, USA), 50 U/mL penicillin and 50 µg/mL streptomycin (15070063, Thermo Fisher Scientific, Inc., Waltham, MA, USA). Cell cultures were cultivated at 37 °C in a humidified atmosphere containing 5% CO_2_ in an incubator (CB150, Binder, Tuttlingen, Germany).

### 4.2. Cell Fixation and Extraction

For cell fixation, 2% paraformaldehyde in serum-free culture medium, supplemented with HEPES (1 mL of 1M HEPES per 50 mL of serum-free medium), was used. The fixation period was ten minutes. Subsequently, the cells were subjected to an extraction-fixation process with cold methanol at −20 °C for a period of five minutes, to enable further immunostaining with antibodies against actin isoforms and other cytoskeletal proteins. For phalloidin staining, the cells were fixed with 2% paraformaldehyde for 20 min and then permeabilized with 0.05% Triton X-100 for a period of five minutes. Following washing with a PBS solution, the cells were stained with antibodies in order to detect cytoskeletal structures in accordance with the established protocols.

### 4.3. Antibodies and Immunofluorescence Microscopy

The following primary antibodies were applied:

- Mouse monoclonal: antibodies anti-pan-actin (clone C4, Cell Signaling Technology, Danvers, MA, USA), anti-α-SM1 (Clone 1A4, IgG2a, AbD Serotec, Kidlington, Oxfordshire, UK), anti-γ-CYA (Clone 2A3, IgG2b, AbD Serotec), anti-β-CYA (Clone 4C2, IgG1, AbD Serotec), anti-γ-SMA (Clone 20D2, IgG1, AbD Serotec), anti-vinculin (Clone hVIN-1, IgG1, Sigma-Aldrich, St. Louis, MO, USA), anti-paxillin (IgG1, Transduction Laboratories, BD Biosciences, San Jose, CA, USA), anti-α-actinin-1 (Clone BM-75.2, IgM, Sigma-Aldrich), anti-desmin (Transduction Laboratories, BD Biosciences), anti-α-tubulin (clone DM1A, IgG1, Thermo Fisher Scientific/Pierce Biotechnology, Rockford, IL, USA), and anti-GAPDH (clone GA1R, IgG, Invitrogen Antibodies, Carlsbad, CA, USA);
- Rabbit monoclonal antibody anti-TAGLN (Clone EPR21215, IgG1, Abcam, Cambridge, UK);
- Rabbit polyclonal antibody anti-SMM (Bio-Rad Laboratories, Hercules, CA, USA).

Phalloidin-Tetramethylrhodamine B isothiocyanate (Phalloidin-TRITC, Sigma-Aldrich, St. Louis, MO, USA) was also used for actin staining.

The secondary antibodies included donkey anti-mouse IgG H&L antibodies (Alexa Fluor^®^ 488, Subclass 2a or Subclass 2b), donkey anti-mouse IgG H&L antibodies (Alexa Fluor^®^ 594, Subclass 1 or Subclass 2b), Alexa Fluor^®^ 488-conjugated IgG H&L and Rhodamine RedTM-X-conjugated donkey anti-mouse IgG H&L antibodies (Jackson ImmunoResearch Laboratories, West Grove, PA, USA).

DAPI (Sigma-Aldrich) for detection of nuclear DNA was added during incubation with secondary antibodies.

Immunofluorescence studies were performed with a Zeiss Axioplan microscope equipped with an Olympus DP70 video camera, using PlanNeofluar 40×/0.75 and 100×/1.3 objectives.

We used a Zeiss LSM900 confocal microscope equipped with a Zeiss Plan-APOCHROMAT 63×/1.4 Oil DIC objective lens (Zeiss, Oberkochen, Germany) for confocal imaging (provided by the Moscow State University Development Program). The laser wavelengths were 405 nm, 488 nm and 561 nm.

### 4.4. RNA Interference

For RNA interference, short hairpin constructs containing 21-nt targeting sequences against β-actin were synthesized and cloned into pLKO.1-puro (Sigma-Aldrich, St. Louis, MO, USA). The sequences used were 5′-CAAATATGAGATGCGTTGTTA-3′ (labelled ‘shβ’ in the images and text), 5′-AATGAAGATCAAGATCATTGC-3′ (labelled ‘shβ2’), and 5′-TAGCATTGCTTTCGTGTAAAT-3′ (labelled ‘shβ3’) (positions 1465–1485, 1056–1076, and 1575–1595 of *ACTB* mRNA, RefSeq NM_001101.5). The most effective construct (5′-CAAATATGAGATGCGTTGTTA-3′) was selected for subsequent experiments. The pLKO.1-shGFP-puro plasmid targeting eGFP (GenBank accession no. U55761) was used as a negative control. Oligonucleotide synthesis and DNA sequencing were performed by Evrogen JSC (Moscow, Russia). The lentiviral DNA constructs, together with the packaging plasmids pΔR8.2 (#12263, Addgene, Watertown, MA, USA) and pVSV-G (#8454, Addgene), were then transfected into human embryonic kidney 293FT packaging cells containing the SV40 large antigen (R70007, Thermo Fisher Scientific), using the TurboFect transfection agent (R0531, Thermo Fisher Scientific). Virus-containing supernatants were collected after 1–2 days of incubation and used to infect fibroblasts in the presence of polybrene (8 µg/mL, Sigma-Aldrich). The infected cell cultures were then incubated for 4–5 days in a medium containing 1 μg/mL puromycin (Sigma-Aldrich). The experiments were performed on days 9–16 after infection.

To address the potential off-target effects of the β-actin-targeting shRNA (5′-CAAATATGAGATGCGTTGTTA-3′), an in silico screen was performed against the human transcriptome. The query set comprised the 21-nt targeting sequence and its reverse complement (5′-TAACAACGCATCTCATATTTG-3′). Searches were carried out with NCBI BLAST (https://blast.ncbi.nlm.nih.gov/Blast.cgi, accessed on 13 March 2026) against the RefSeq RNA database, and the results were restricted to homo sapiens. BLAST hits were filtered by alignment length and mismatch count using two thresholds: a stringent filter (≥19 nt aligned with ≤2 mismatches) and a broader screen (≥17 nt aligned with ≤6 mismatches). Using the broader criteria (excluding *ACTB*), two candidate off-target genes with potential relevance to differentiation were identified. For *RCOR3* (REST corepressor 3; GeneID 55758), two predicted transcript isoforms (XM_054337625.1 and XM_047425038.1) had a perfect 18-nt match (positions 4–21 of the 21-nt query; 18/18; zero mismatches; 100% identity). For *TBX20* (T-box transcription factor 20; Gene ID 57057), two predicted transcript isoforms (XM_017012456.2 and XM_054358685.1) showed 19-nt alignments (positions 2–20) with two mismatches (19 nt; two mismatches; 89.47% identity). Analysis of our RNA-seq data revealed that the transcript levels of *RCOR3* and *TBX20* remained unchanged following β-actin knockdown, suggesting that they are not involved in mediating the observed phenotype.

### 4.5. Western Blotting

Cell culture extracts were prepared by scraping the cells into ice-cold Lysis-M buffer (4719964001, Roche Diagnostics Corporation, Indianapolis, IN, USA), containing protease inhibitors (04693116001, Roche Diagnostics Corporation) and phosphatase inhibitors (04906837001, Roche Diagnostics Corporation). After 15 min of incubation on ice, the sample was centrifuged at 11,000× *g* for 10 min at +4 °C. The protein concentration in cell lysates was measured using the Bradford method. Protein amounts ranging from 5 to 20 μg were separated on an 8–12% polyacrylamide gel with SDS and transferred to a PVDF membrane (Millipore, Billerica, MA, USA). The membranes were blocked in SuperBlock Blocking Buffer (Thermo Fisher Scientific) for 15–20 min or in a 5% solution of skim milk powder for 1–1.5 h at room temperature, then stained with specific antibodies for 1 h at room temperature. The membranes were washed three times for 5 min with ice-cold PBS solution with Tween-20 (PBS-T, 0.1%) on a shaker, then incubated in secondary antibodies for 30 min and washed again three times for 5 min with ice-cold PBS-T solution. After washing, following preliminary drying, the desired bands were detected using enhanced chemiluminescence WesternBright^TM^ Quantum detection kit (Advansta Inc., San Jose, CA, USA).

Densitometric analysis of images was performed using TotalLab software (version 1.11). Antibodies to the total actin (pan-actin), α-tubulin or GAPDH were used as a loading control. Protein levels were estimated by analyzing the results of at least three independent experiments.

### 4.6. RNA Sequencing

For the next-generation sequencing (NGS) two groups of samples were used: control gingival fibroblasts and gingival fibroblasts expressing shRNA targeting β-actin. Total RNA was isolated from control and β-actin knockdown cells using the QIAGEN RNeasy Kit (QIAGEN, Hilden, Germany), following the manufacturer’s protocol. RNA concentration was quantified using the Agilent RNA 6000 Nano Qubit RNA Assay Kit (Thermo Fisher Scientific, USA). Subsequently, mRNA enrichment and library preparations for sequencing were carried out using the KAPA RNA Hyper RiboErase Kit (KAPA Biosystems, Wilmington, MA, USA). Concentration and quality assessment of the resulting libraries were performed using the Qubit RNA HS Assay Kit (Life Technologies, Carlsbad, CA, USA) and the Agilent Tapestation instrument (Agilent Technologies, Santa Clara, CA, USA). Sequencing was performed on an Illumina NextSeq 550, generating at least 20 million raw reads per sample with a read length of 75 bp. For each sample, RNA sequencing reads were aligned to the H. sapiens UCSC hg38 reference genome (GRCh38). Read counts per gene were normalized using the DESeq2 software package (DOI: 10.18129/B9.bioc.DESeq2), version 1.38.1 (https://bioconductor.org/packages/release/bioc/html/DESeq2.html (accessed on 17 April 2024)) enabling the calculation of differential gene expression based on these adjusted values.

### 4.7. PCR Analysis

Total RNA was extracted from the analyzed cells using the TRIzol reagent (Invitrogen, Carlsbad, CA, USA), and RNA concentration was determined using a NanoDrop 1000 spectrophotometer (Thermo Fisher Scientific). For cDNA synthesis, 1 μg of total RNA was reverse transcribed using M-MLV reverse transcriptase (Promega). Real-time quantitative PCR was conducted on the CFX96 Touch Real-Time PCR Detection System (Bio-Rad Laboratories, Hercules, CA, USA) with 5X qPCRmix-HS SYBR (Evrogen, Moscow, Russia). All PCR reactions were run in triplicate, with *RER1* (Retention in Endoplasmic Reticulum Sorting Receptor 1) serving as the normalizer. Relative expression of the genes was estimated by the 2−ΔΔCt method. Gene-specific primers were synthesized by Evrogen, and are presented in the Table 1. The resulting end-point PCR products were separated electrophoretically in a 2% agarose gel.

### 4.8. Morphometric Analysis

The IF microscopy images were analyzed using ImageJ Fiji v1.53u software, then processed and presented using Adobe Photoshop version 22.4.2. ImageJ Fiji v1.53u was used to manually trace cell outlines in order to measure the area occupied by cells on the substrate, circularity, and quantify the mean fluorescence intensity of cytoskeletal proteins per cell. The ‘Set Measurements’—‘Area’ and ‘Circularity’ tools were used to measure cell area and circularity. The ‘Set Measurements’—‘Mean Grey Value’ tools were used for the fluorescence intensity quantification.

The analysis of FAs was performed using paxillin immunostaining. After splitting the IF channels and thresholding, for each cell three square areas with dimensions of 10 × 10 μm (100 μm^2^) within the lamellar zone except the cell edge were selected and the mean of FA amount was calculated. We quantified the number of paxillin-positive puncta > 0.2 µm^2^, excluding peripheral FAs. The FA area was measured using the ‘Analyze particles’ tool for paxillin-positive puncta > 0.2 µm^2^.

### 4.9. Statistical Analysis

Statistical analysis was performed using GraphPad Prism 9. The data are presented as the mean ± standard error of the mean (SEM), calculated from the results of at least three independent experiments. At least 20 fields of view were analyzed for each biological replicate for immunofluorescence intensity, morphometric and FA measurements. The results were analyzed using the Mann–Whitney U or t-test for statistical evaluation. Values of *p* < 0.001 (***), *p* < 0.01 (**) and *p* < 0.05 (*) were considered statistically significant.

## Figures and Tables

**Table 1:** Forward and Reverse Primer PCR Sequences for PCR analysis.

Primers	Sequence of Nucleotides (nt)	Size (nt)
*TUBA1A*	Forward 5′–GTTGGTCTGGAATTCTGTCAG–3′ Reverse 5′–AAGAAGTCCAAGCTGGAGTTC–3′	21 21
*ACTA2*	Forward 5′–CTATGAGGGCTATGCCTTTGCC–3′ Reverse 5′–GCTCAGCAGTAGTAACGAAGGA–3′	21 22
*ACTB*	Forward 5′–ACAGAGCCTCGCCTTTGC–3′ Reverse 5′–GAGGCGTACAGGGATAGCAC–3′	18 20
*ACTG1*	Forward 5′–CAAAAGGCGGGGTCGCAA–3′ Reverse 5′–TGGGGTACTTCAGGGTCAGG–3′	18 20
*ACTG2*	Forward 5′–CATGTACGTCGCCATTCAAGC–3′ Reverse 5′–TTGATGTCTCGCACAATTTCTCT–3′	21 23
*MYL9*	Forward 5′–ACCAGACCGCAGATCTGATC–3′ Reverse 5′–TCACCCAGTGTGACAAGAACA–3′	20 21
*VCL/MVCL*	Forward 5′–CCGCTGAGGTGGGTATAGGT–3′	20
	Reverse 5′–TGGTAGCTTCCCGATGCAAG–3′	20
*CALD1*	Forward 5′–GTGACCAACCAGAAGGCTCA–3′	20
	Reverse 5′–CTTCGAGTCGCATCTCCTCC–3′	20
*TAGLN*	Forward 5′–CTGTCCGAACCCAGACACAA–3′	20
	Reverse 5′–CAGCCAATGCACTCACAAGG–3′	20
*RER1*	Forward 5′–ACCCACCAAACAGAACGAGG	20
	Reverse 5′–AGAACACCGGGACGTTGAAA	20

## Data Availability

The original contributions presented in this study are included in the article; further enquiries can be directed to the corresponding author.

## Supplementary Materials

The following supporting information can be downloaded at https://www.mdpi.com/article/10.3390/ijms27135820/s1.
