# Supplementary material for: Induction of Smooth Muscle Differentiation in Fibroblasts by Modulation of Cytoplasmic Actin Ratio
Source: Int J Mol Sci. 2026 Jun 27;27(13):5820. doi: 10.3390/ijms27135820 (PMC13361686; doi:10.3390/ijms27135820)
Supplement: Supplementary file 1 [file ijms-27-05820-s001.zip › Supplementary Figures S1 and S2.pdf]

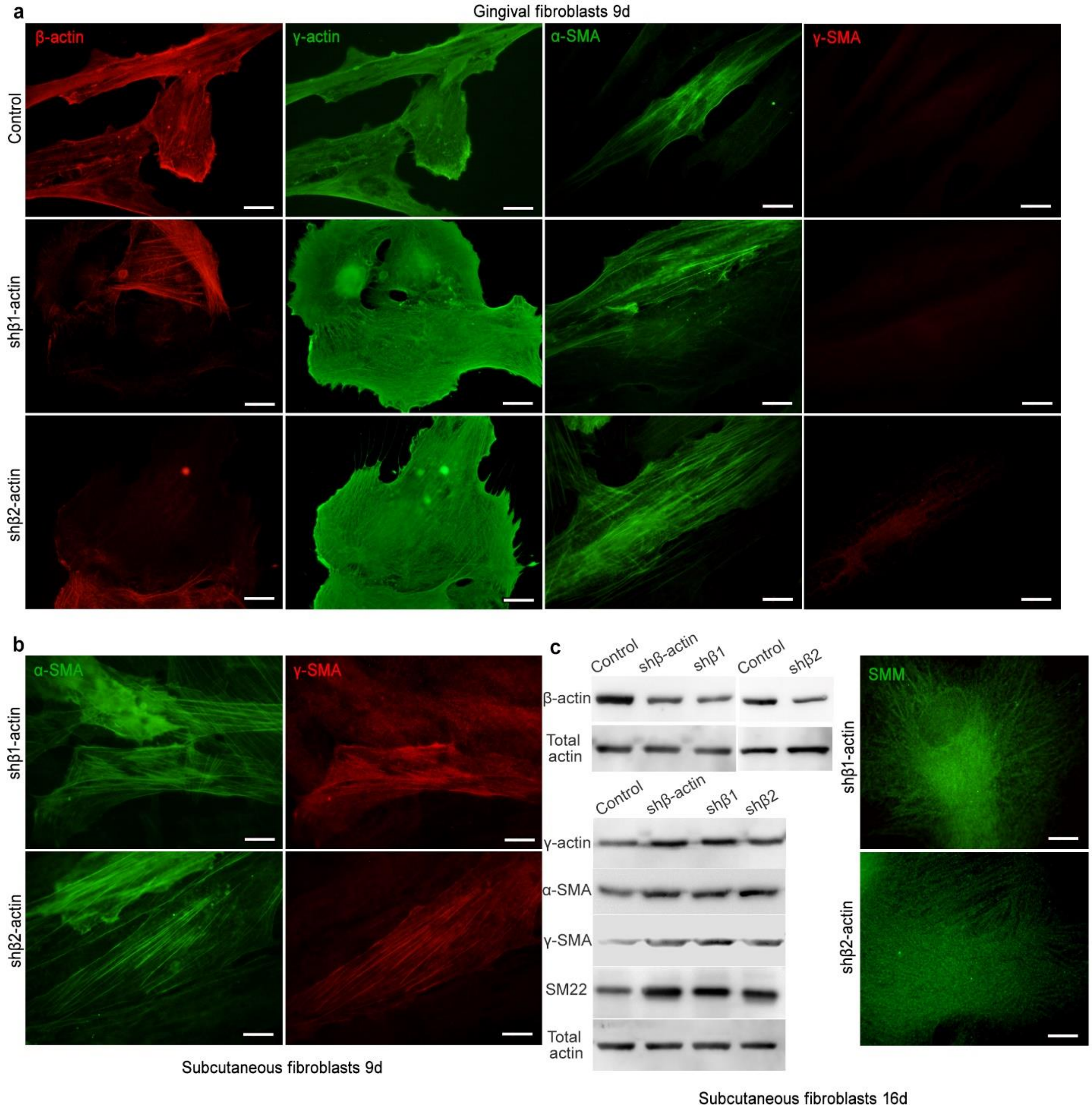

Supplementary Figure 1. Smooth muscle actins and smooth muscle cell markers in human fibroblasts after β-actin suppression. Alternative short hairpin constructs containing 21-nt targeting sequences against β-actin were used. **(a)** Representative immunofluorescence images of β-actin, γ-actin, α-SMA, and γ-SMA in gingival fibroblasts 9 days after transduction with control, shβ1-actin, or shβ2-actin constructs; **(b)** Representative immunofluorescence images of α-SMA and γ-SMA in subcutaneous fibroblasts 9 days after transduction with shβ1-actin or shβ2-actin constructs; **(c)** Representative western blot analysis of β-actin, γ-actin, α-SMA, γ-SMA, SM22, and total actin, together with representative immunofluorescence images of smooth muscle myosin (SMM) in subcutaneous fibroblasts 16 days after β-actin suppression with alternative short hairpin constructs. Scale bars: 15 μm.

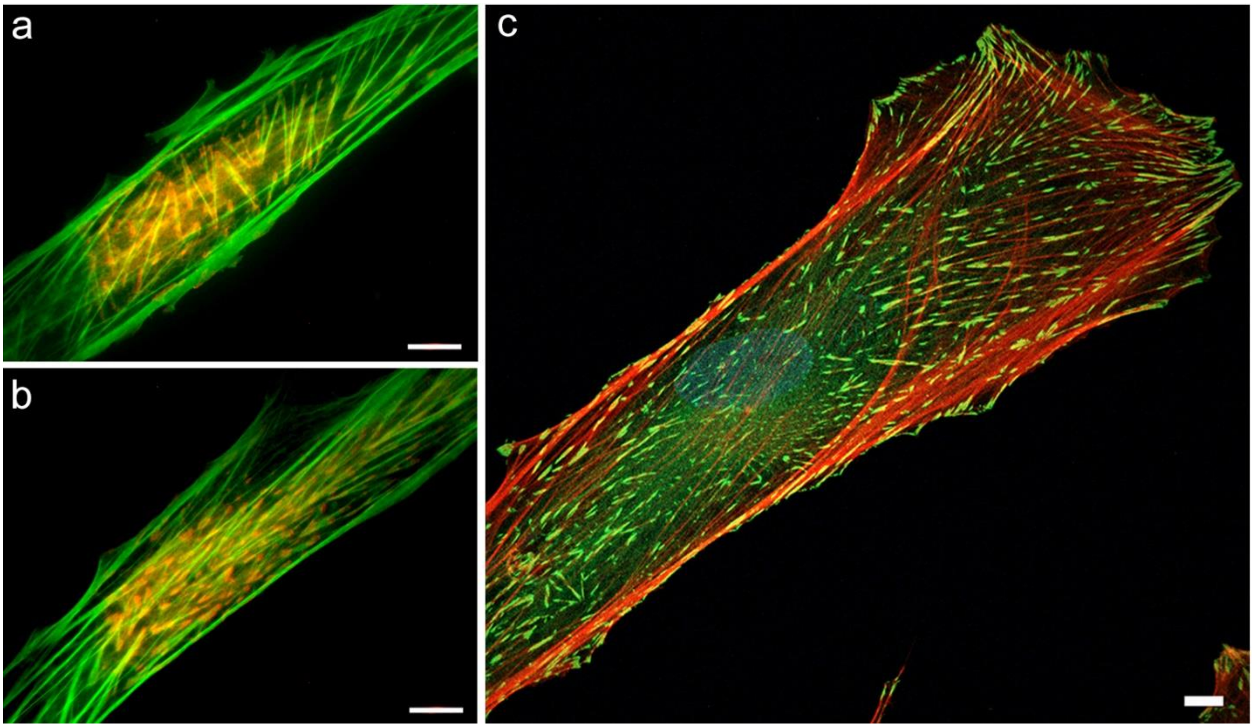

Supplementary Figure 2. Human subcutaneous fibroblasts after β-actin suppression. Representative images of elongated cells observed 16 days after transduction with shRNA targeting β-actin. **(a,b)** Immunofluorescence microscopy of α-SMA (green) and paxillin (red); **(c)** Confocal immunofluorescence microscopy of F-actin (phalloidin, red), paxillin (green), and DAPI (blue). Scale bars: 5 μm.
