# Supplementary material for: Induction of Smooth Muscle Differentiation in Fibroblasts by Modulation of Cytoplasmic Actin Ratio
Source: Int J Mol Sci. 2026 Jun 27;27(13):5820. doi: 10.3390/ijms27135820 (PMC13361686; doi:10.3390/ijms27135820)
Supplement: Supplementary file 1 [file ijms-27-05820-s001.zip › Supplementary WB materials.pdf]

Original blot images corresponding to representative panels

Figure 2d

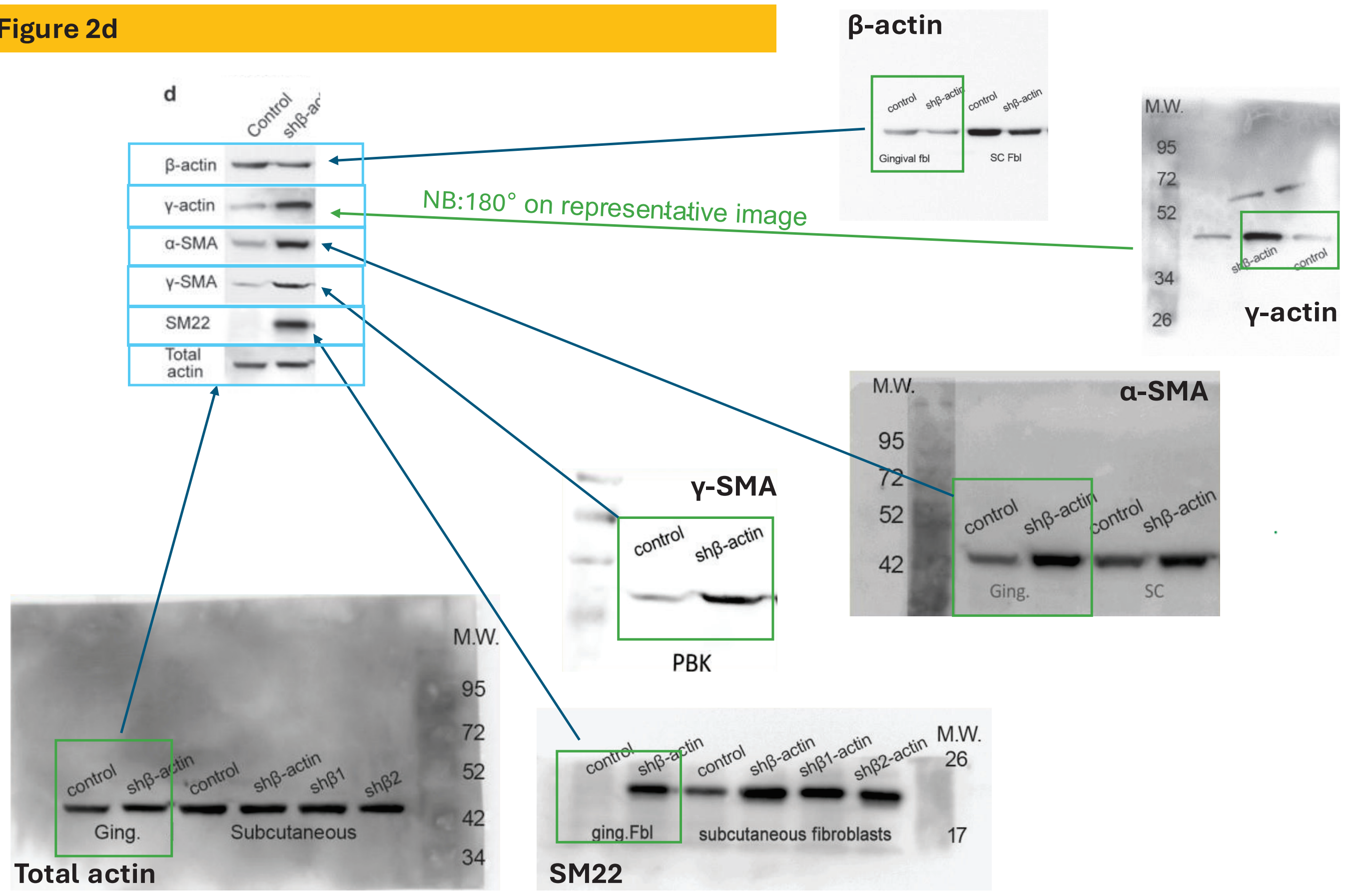

Figure 2e

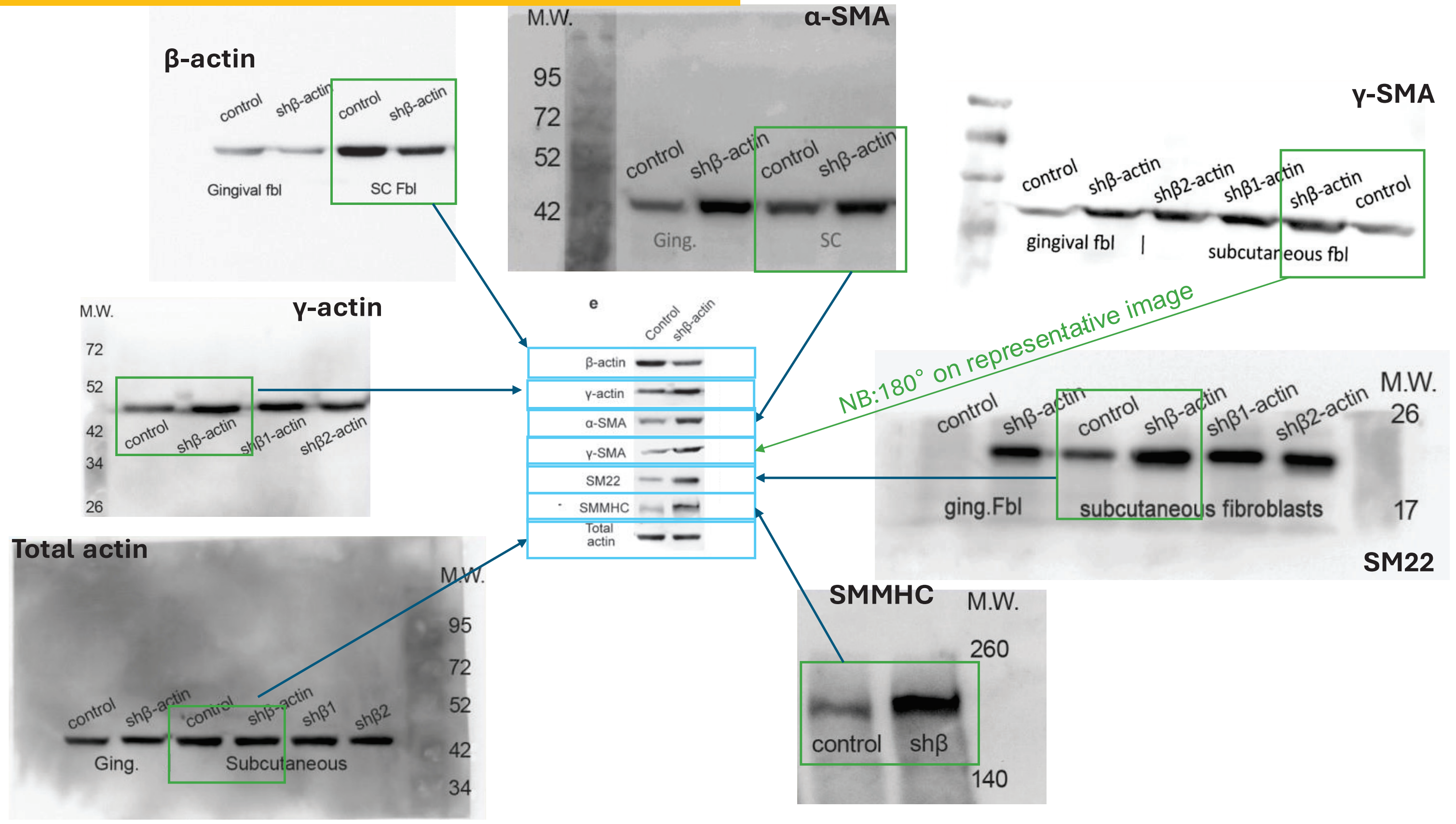

Figure 2 Additional loading controls for WB from Figure 2

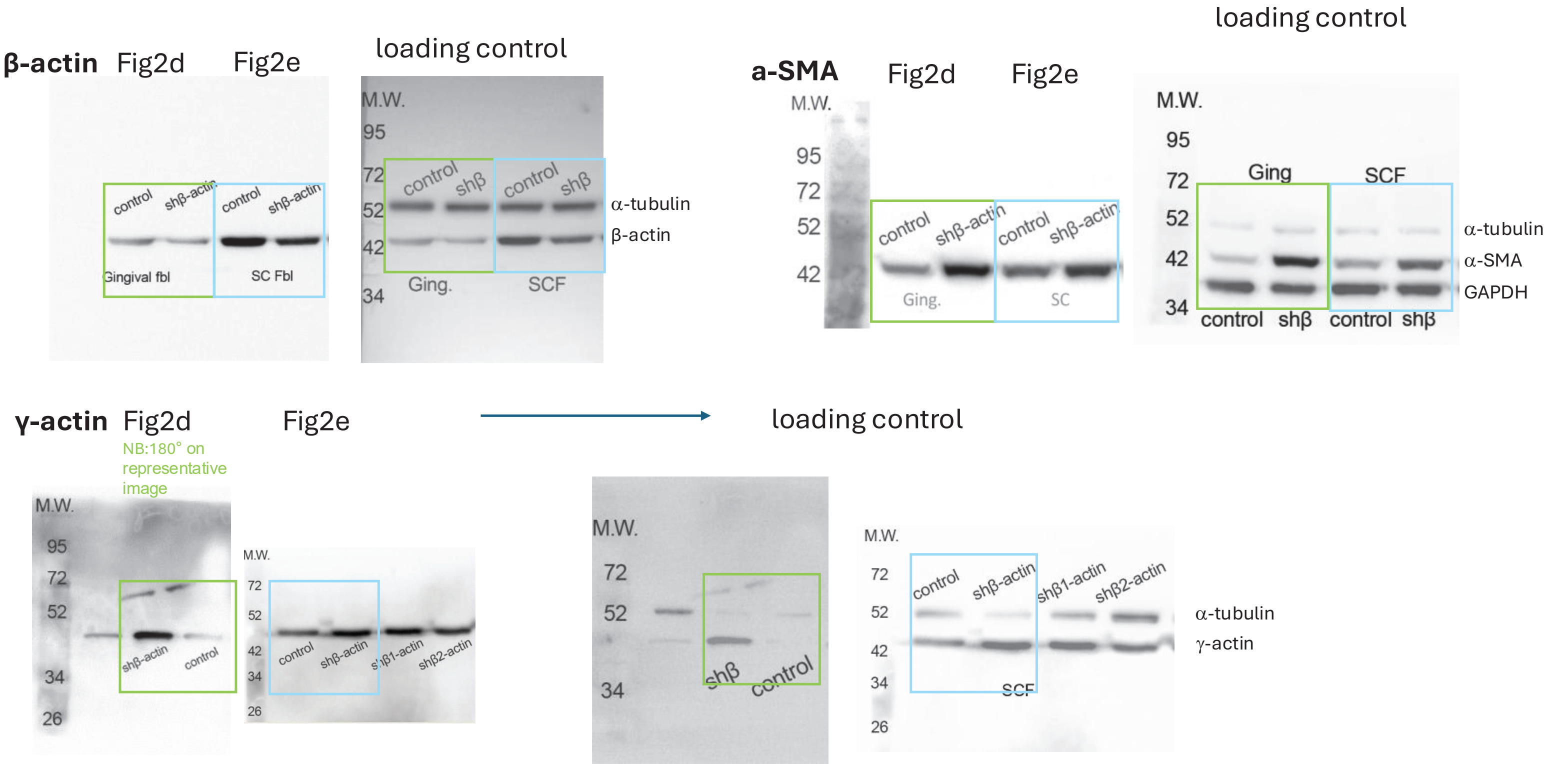

Figure 2 Additional loading controls for WB from Figure 2

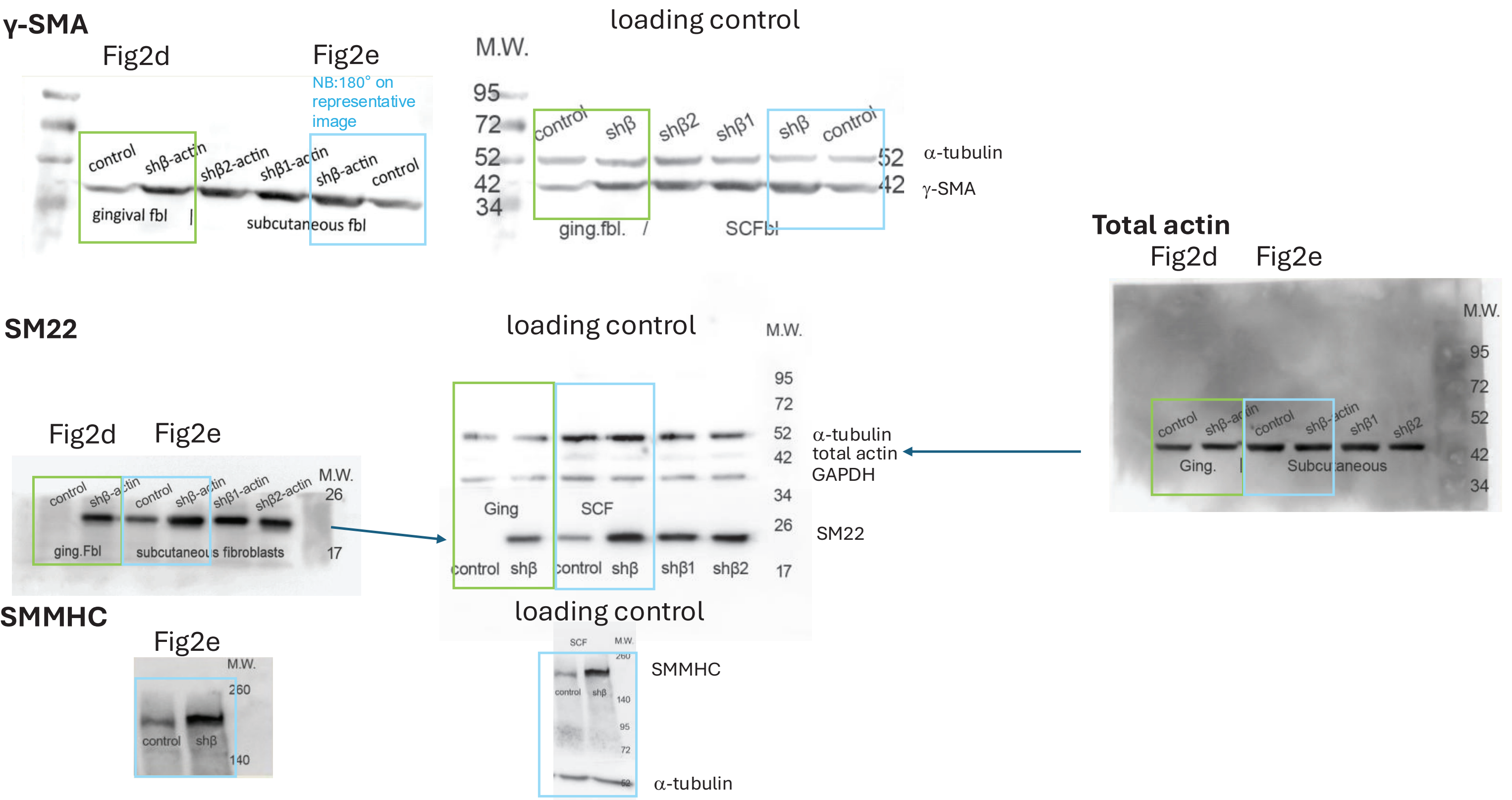

Figure 3

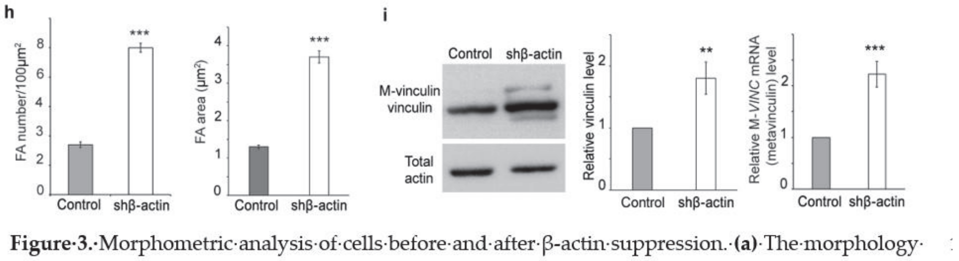

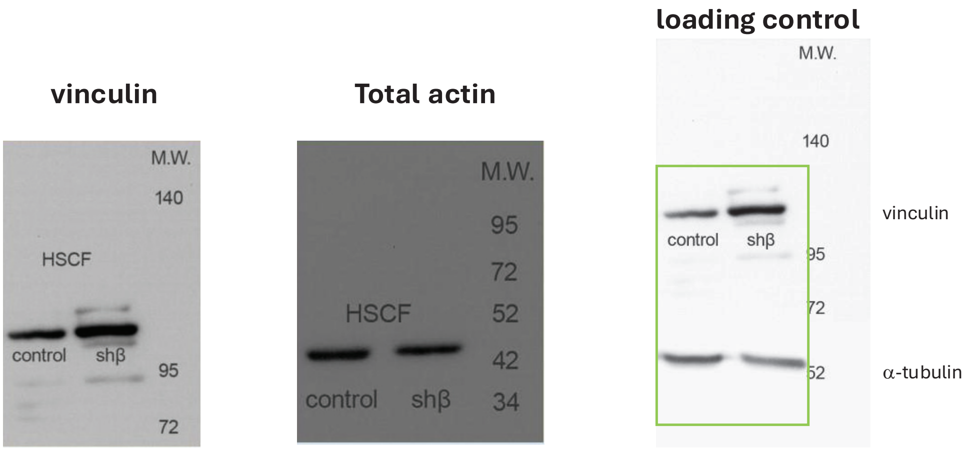

Supplementary Figure S1

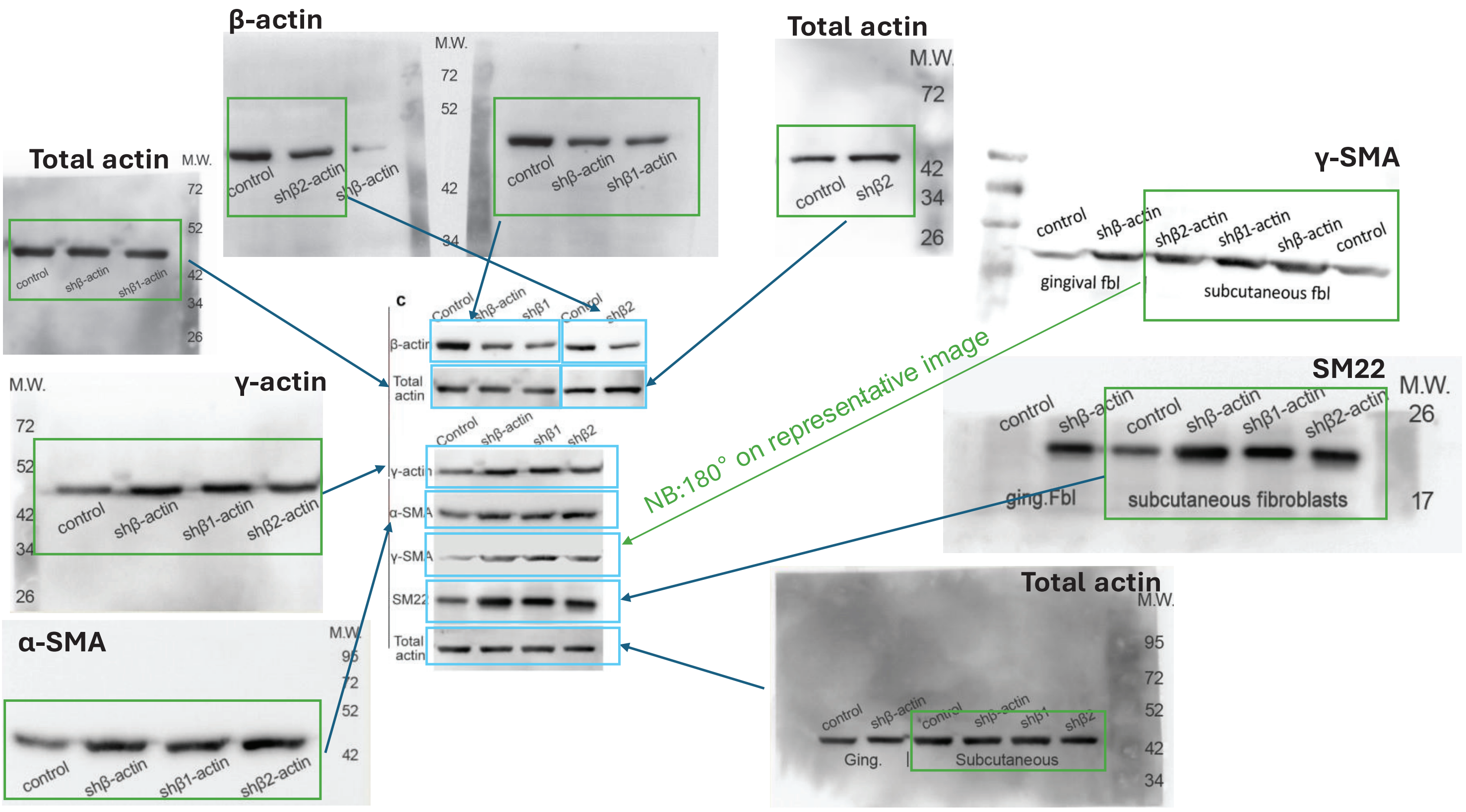

Supplementary Figure S1 Additional loading controls for WB from Supplementary Figure S1

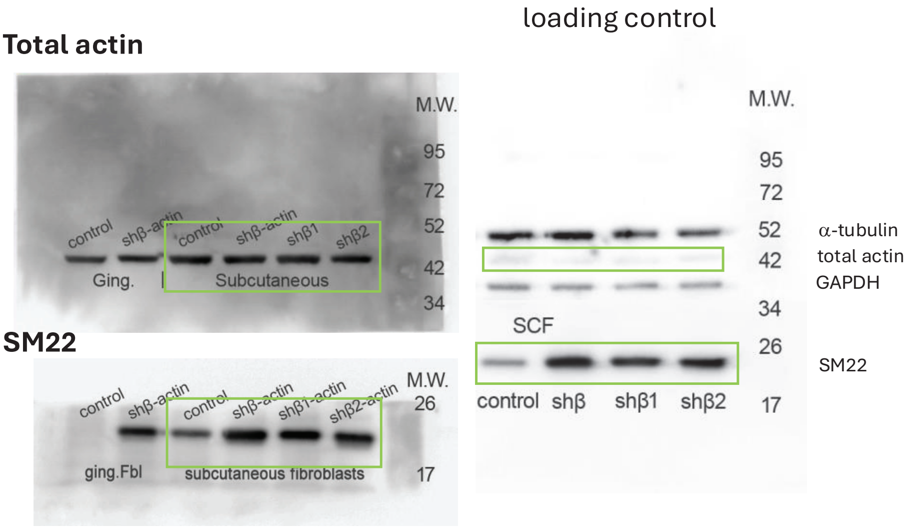

Supplementary Figure S1 Additional loading controls for WB from Supplementary Figure S1

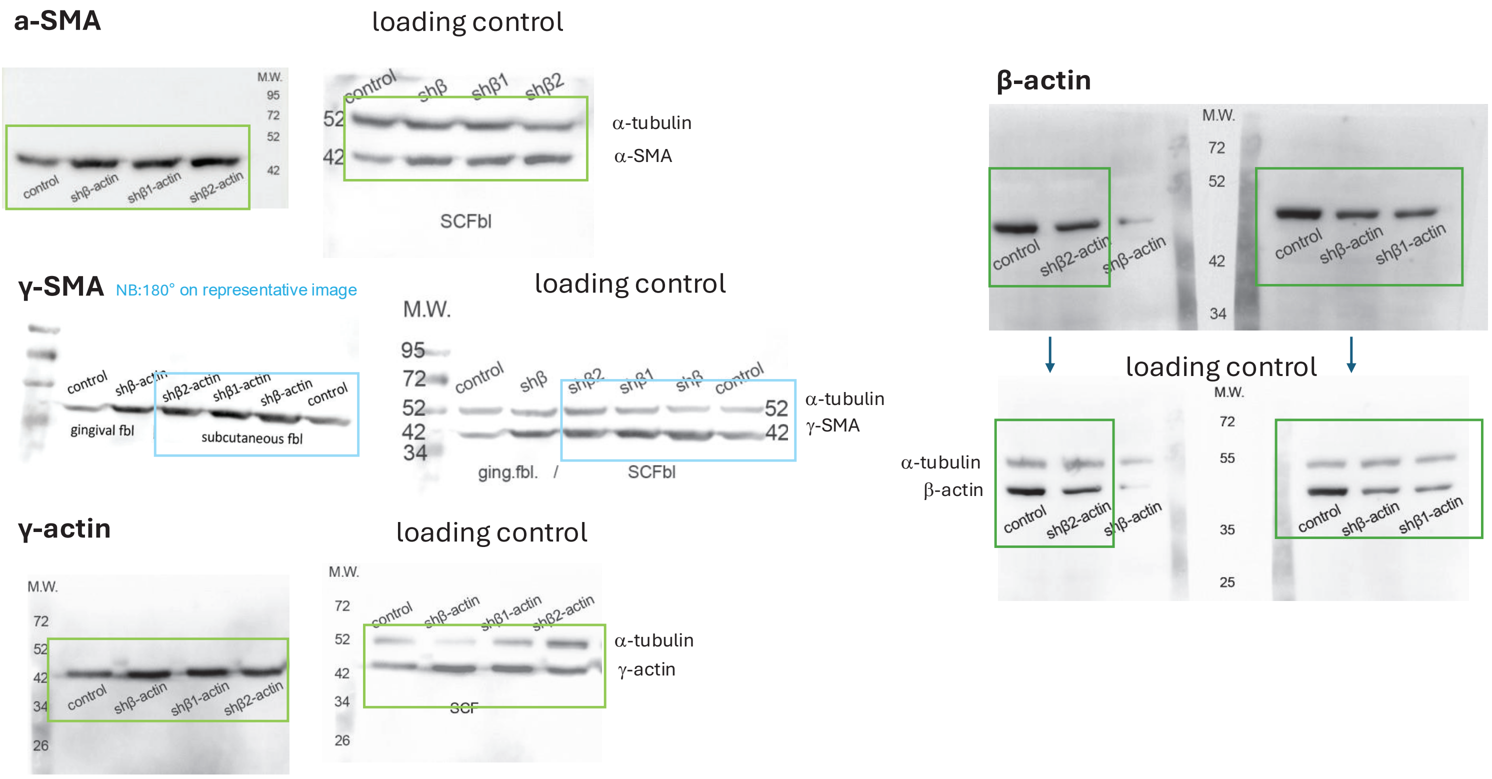

Original blot images for additional replicates

protein	experiment 2 (or 2, 3)	experiment 3 (or 4)
α–SMA<br>α–tubulin<br>Gingival fibroblasts	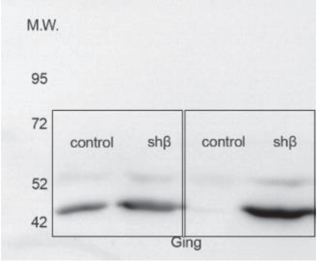	
α–SMA<br>α–tubulin<br>Subcutaneous fibroblasts	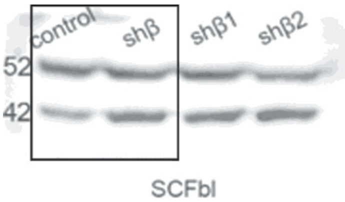	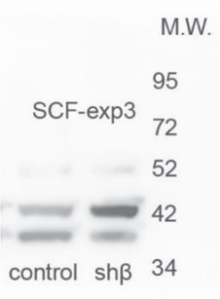
β-actin<br>α–tubulin<br>Gingival fibroblasts	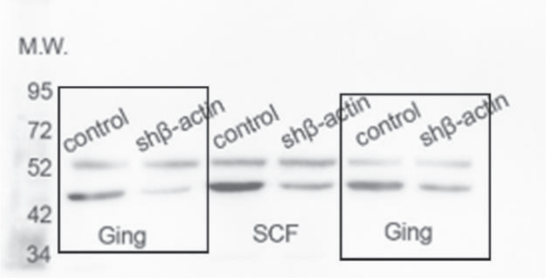	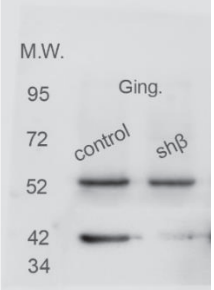
β-actin<br>α–tubulin<br>Subcutaneous fibroblasts	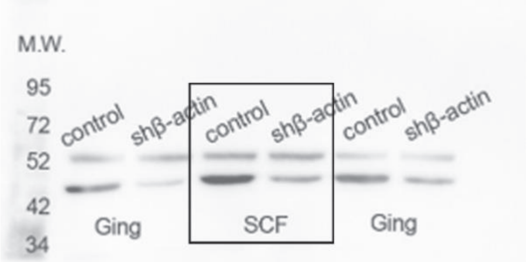	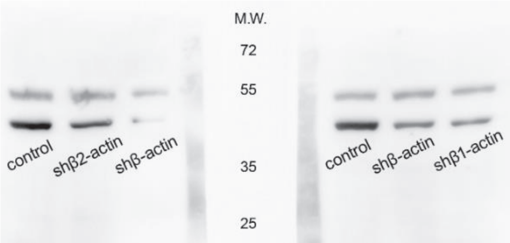
γ-actin<br>α–tubulin<br>Gingival fibroblasts	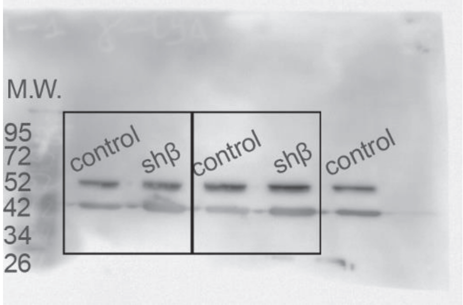	
γ-actin<br>α–tubulin<br>Subcutaneous fibroblasts	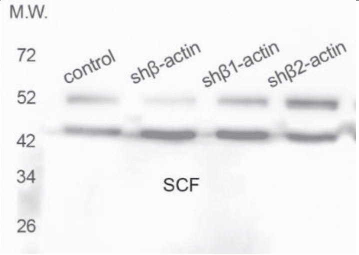	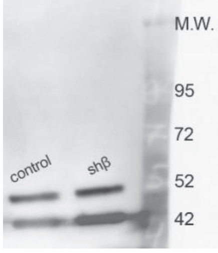

<table>
<tr><td>SM22
GAPDH (exp2)
or SM22
Total actin
α–tubulin
(exp3)
Gingival
fibroblasts</td><td>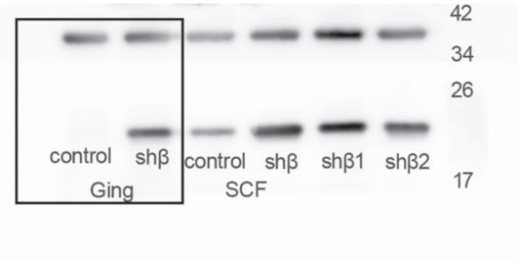
</td><td>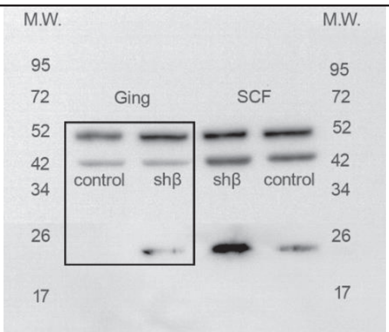
</td></tr>
<tr><td>SM22
GAPDH (exp2)
or SM22
Total actin
α–tubulin
(exp3)
Subcutaneous
fibroblasts</td><td>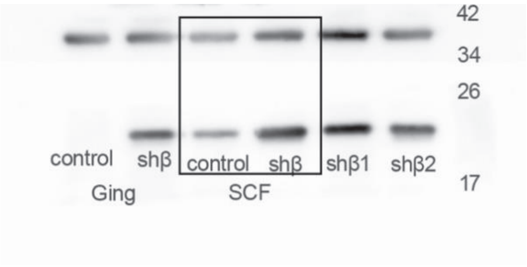
</td><td>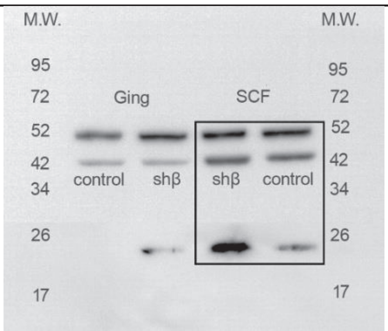
</td></tr>
<tr><td>SMM
α–tubulin
Subcutaneous
fibroblasts</td><td>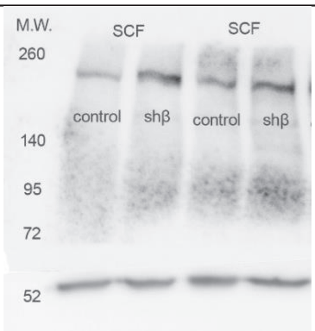
</td><td></td></tr>
<tr><td>vinculin
α–tubulin
Subcutaneous
fibroblasts</td><td>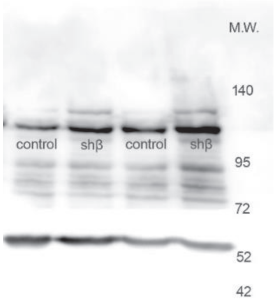
</td><td>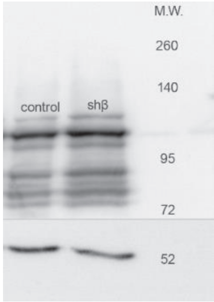
</td></tr>
<tr><td>γ–SMA
α–tubulin
Gingival
fibroblasts</td><td>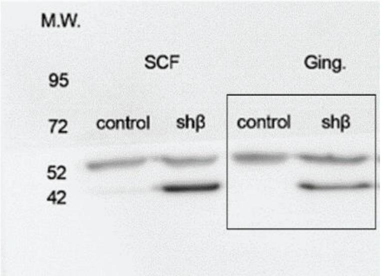
</td><td>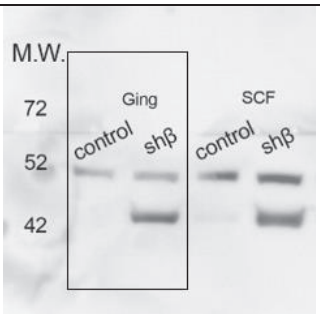
</td></tr>
<tr><td>γ–SMA
α–tubulin
Subcutaneous
fibroblasts</td><td>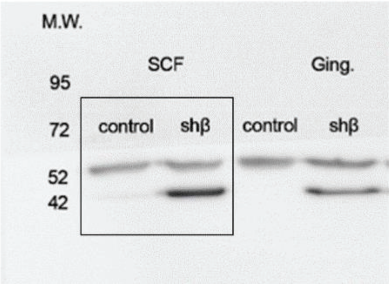
</td><td>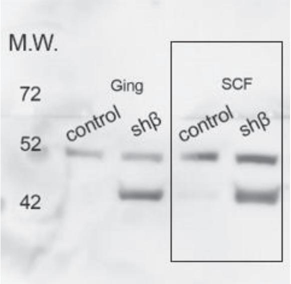
</td></tr>
</table>
