# Supplementary figures and images for: Induction of Smooth Muscle Differentiation in Fibroblasts by Modulation of Cytoplasmic Actin Ratio
Source: Int J Mol Sci. 2026 Jun 27;27(13):5820. doi: 10.3390/ijms27135820 (PMC13361686; doi:10.3390/ijms27135820)

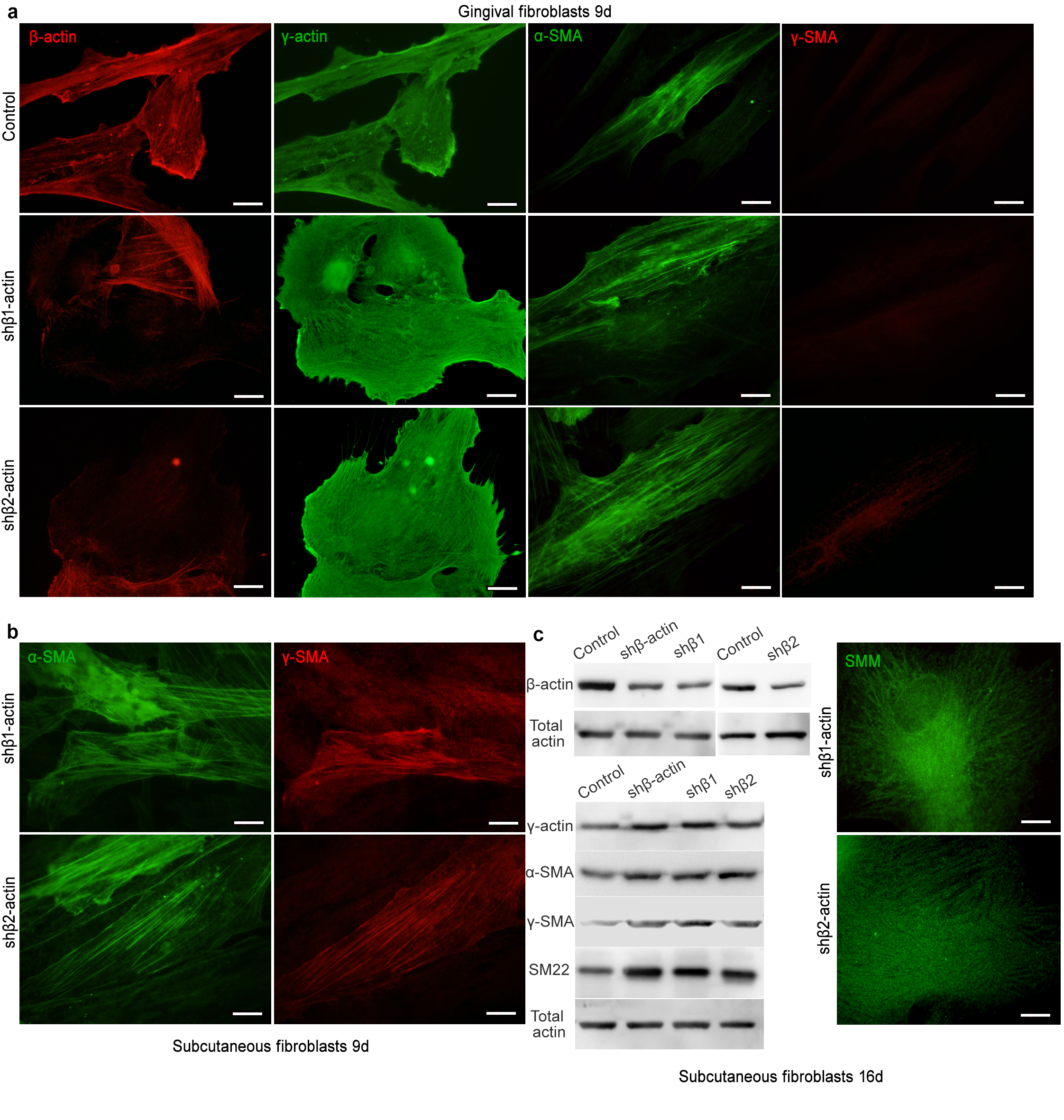

Supplement: Supplementary file 1 [file ijms-27-05820-s001.zip › Supplementary Figure S1.jpg]

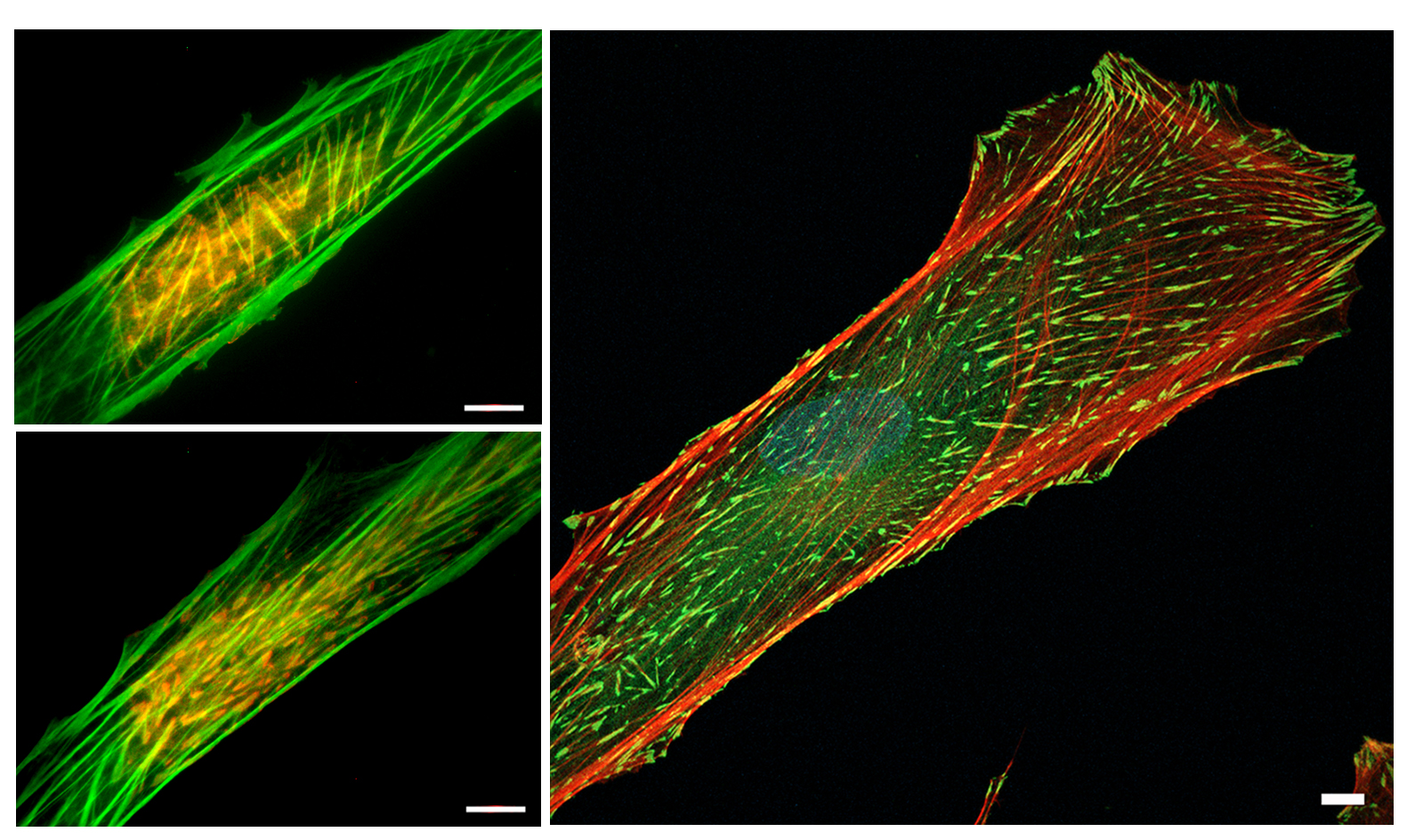

Supplement: Supplementary file 1 [file ijms-27-05820-s001.zip › Supplementary Figure S2.jpg]
